# A Remarkable Case

**Published:** 1880-03

**Authors:** 


### ARTICLE VII.

### *A Remarkable Case.*

On the 6th December, 1878, a gentleman called and requested me to attend his wife at his residence, as she had for some time past complained of difficulty in swallowing, produced by artificial teeth. On examining the patient's mouth, I found the tongue raised considerably upwards and backwards by a mass of mucous tissues almost as large as the tongue itself, and giving very much the appearance of a second tongue, that is to say, one under the other, only the lower being shapeless. On examining further I found the patient wearing a complete upper plate of gold and vulcanite with springs; these springs I found attached to a lower gold plate on which was one molar tooth on each side, but I could not see nor could I feel any bar of gold or other material connecting the two teeth. I must mention that owing to this enormous mass of tissues, it was almost impossible to see anything whatever; however, in trying to raise the lower plate I found I could not do so, and, on a still closer examination, I discovered that the whole of the gold bar which usually rests against the back of the front teeth was entirely buried and grown over by a firm fibrous band of tissues. I asked my patient a few questions, and found she had not had her plates out of her mouth for *five years*, and that the difficulty in swallowing had gradually increased for a long time. I then told her I should be obliged to cut the lower plate out, and left to get the necessary instruments.

The same afternoon I called, accompanied by Mr. Willis to assist me. I first cut both springs through close to the lower swivels, and so removed the upper plate in order to get as much room as possible. Mr. Willis then held one end of the lower plate firmly by the swivel-head with a pair of pliers, while I endeavored to cut through the fibrous bands with a straight bistoury, meaning to cut on the gold bar itself for safety; but this I found I could not do, my knife having a great tendency to slip, and it being very difficult to keep the bulging mass of mucous tissues out of the way. I then took a curved bistoury, and with that succeeded in cutting by degrees through the band, Mr. Willis keeping the plate firmly raised the whole time, and so to say following me up each cut I gave.

The fibrous nature of the band was clear, from the firmness with which the plate was bound down, and also from the peculiar sound produced each time the knife was used. I should say that this band was quite one-eighth of an inch in thickness, and extended from the right molar to the left molar teeth. I remained with my patient some time to see that no hemorrhage occurred, and after prescribing a little carbolic acid liq. potassæ as a mouth-wash, left.

Everything went on well until the 9th of December (three days after the plate was cut out,) when my patient complained of the last lower tooth on each side cutting her tongue and causing pain in swallowing. As these were both mere shells with very rough edges, I removed them, and from that time everything was satisfactory.

On the 18th of December I put in a new upper suction plate of celluloid, but I advised her not to wear any lower piece for a long time, not until all the abnormal growth of tissues had subsided, which I should say would take many months.

I saw this lady's husband about two months ago, and he then said she was perfectly comfortable, but as I have not seen her recently myself I cannot say whether the parts have assumed their normal character or not.

What strikes one as being so marvelous, and which makes these cases so rare, is that anyone can be found who could endure the pain that must occur. We know how painful even a small ulcer is; but in this case the whole gold bar must have ulcerated right under the tongue and then the two ulcerated surfaces united. Yet I could not get my patient to admit that she had suffered any extraordinary pain.

There is only one other case that I know of similar to this, and that occurred in Mr. Turner's practice; I think Mr. Moon also has seen a case something similar.—*Mr. Canton, in Transactions of Odont. Soc. of Great Britain.*

---

